# Tension Pneumothorax: Is it Sarcoma or Pazopanib?

**DOI:** 10.7759/cureus.10945

**Published:** 2020-10-14

**Authors:** Vinoja Sebanayagam, Samer Alkassis, Bayan Alshare, Neelima Thati

**Affiliations:** 1 Hematology and Oncology, Wayne State University School of Medicine, Detroit, USA; 2 Internal Medicine, Wayne State University School of Medicine, Detroit, USA

**Keywords:** synovial sarcoma, trapezius, pazopanib, pneumothorax

## Abstract

Synovial sarcomas are rare malignant tumors that originate from primitive pluripotent mesenchymal stem cells that look similar to the developing synovium, but are histologically unrelated to it. Sarcomas commonly metastasize to the lungs and surrounding pleura, with a documented incidence as high as 85% for pleural-based metastases. The incidence of spontaneous pneumothorax in patients with sarcomas is only 1.9%, with synovial sarcoma being the third most common type of sarcoma associated with pneumothorax. While surgical resection is usually the treatment for localized primary synovial cell sarcoma, metastatic disease requires systemic therapy, mainly chemotherapy. Failure of chemotherapy calls for the use of targeted therapeutic agents such as pazopanib. Pazopanib has been linked to the incidence of spontaneous pneumothorax in previous case studies. However, primary research fails to establish a statistically significant causal association. Research shows that pneumothorax can result from lung metastases independent of therapeutic side effects. We report a case of synovial sarcoma of trapezius origin with secondary lung metastases, and development of pneumothorax after pazopanib treatment. We discuss the incidence of pneumothorax as a medication side effect versus independent effect of natural disease progression, and how this plays role in deciding when to continue using a medication in the face of complications.

## Introduction

Synovial cell sarcomas are rare malignant tumors that originate from primitive pluripotent mesenchymal stem cells that microscopically resemble the developing synovium, but are unrelated to the synovial tissue [1,2]. They account for 8%-10% of all soft tissue sarcomas, with 85% occurring in extremities [3], and have a higher propensity for lower limbs [1,4]. The incidence of synovial sarcomas is higher among men than women [4] and adults who are in their third to fifth decade of life [1].

No optimal treatment for synovial sarcomas has been defined so far [2]. Complete surgical resection with negative margins is the widely used first-line treatment for primary synovial sarcoma without metastases [2]. The use of systemic chemotherapeutic agents such as doxorubicin and ifosfamide in the management of synovial sarcoma is controversial, however, it has been used in many cases reported in the literature [5-7], and is thought to help control distant metastases of synovial sarcoma [2]. In instances where patients fail chemotherapeutic agents, targeted therapeutic agents such as pazopanib are employed to treat metastatic soft tissue sarcomas [8]. In this article, we report a case of synovial sarcoma of the trapezius, the approach we used to manage it and sequelae of disease and treatment. We focus on the presumed complications of pazopanib therapy, which was used to treat our patient, in comparison to risks associated with disease progression in the absence of treatment.

## Case presentation

A 28-year-old female was diagnosed in Tunisia with stage IV synovial sarcoma originating from the soft tissue of the left trapezius with lung metastases, after complaining of shoulder swelling and pain back in July 2018. Written reports of the CT imaging obtained in Tunisia revealed that she had a 4 x 2 cm left paravertebral pleural formation, 1 cm left pleural nodule and 10 x 9 x 3.5 cm left posterior dorsal wall mass of muscular origin presumed to be the trapezius, at the time of diagnosis. Outside reports of the biopsy of the intramuscular mass revealed that she was diagnosed with a grade 2 monophasic synovial cell sarcoma. She had no family history of malignancy. She received 6 cycles of adriamycin and ifosfamide with mesna. The size of tumor decreased after the third cycle and continued to be stable afterwards. The plan was to start radiation after completion of chemotherapy, but she relocated to the United States before starting radiation therapy.

**Figure 1:**
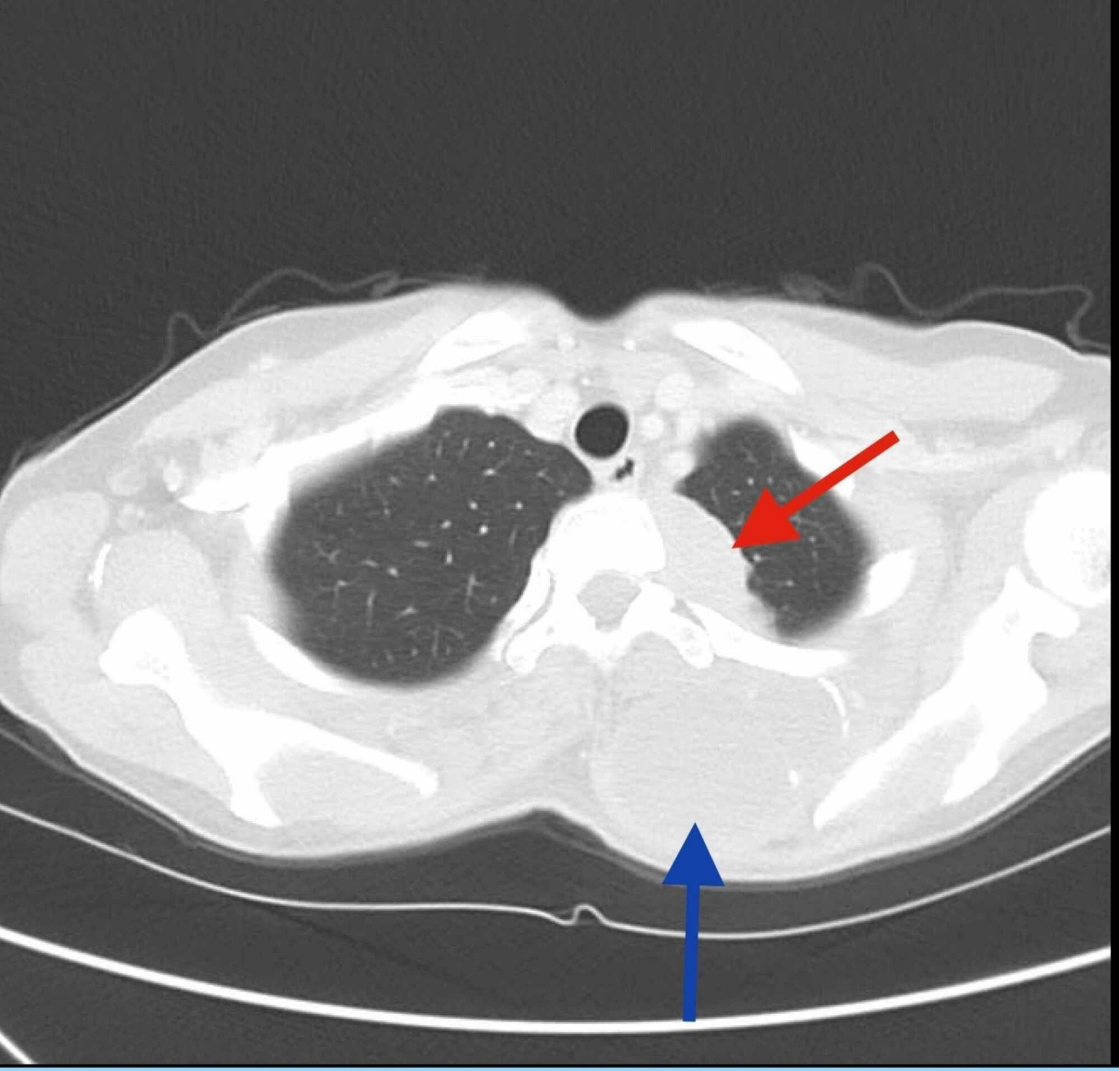
CT scan of axial section showing left pleural nodule (red arrow) and left trapezius muscle mass (blue arrow)

**Figure 2:**
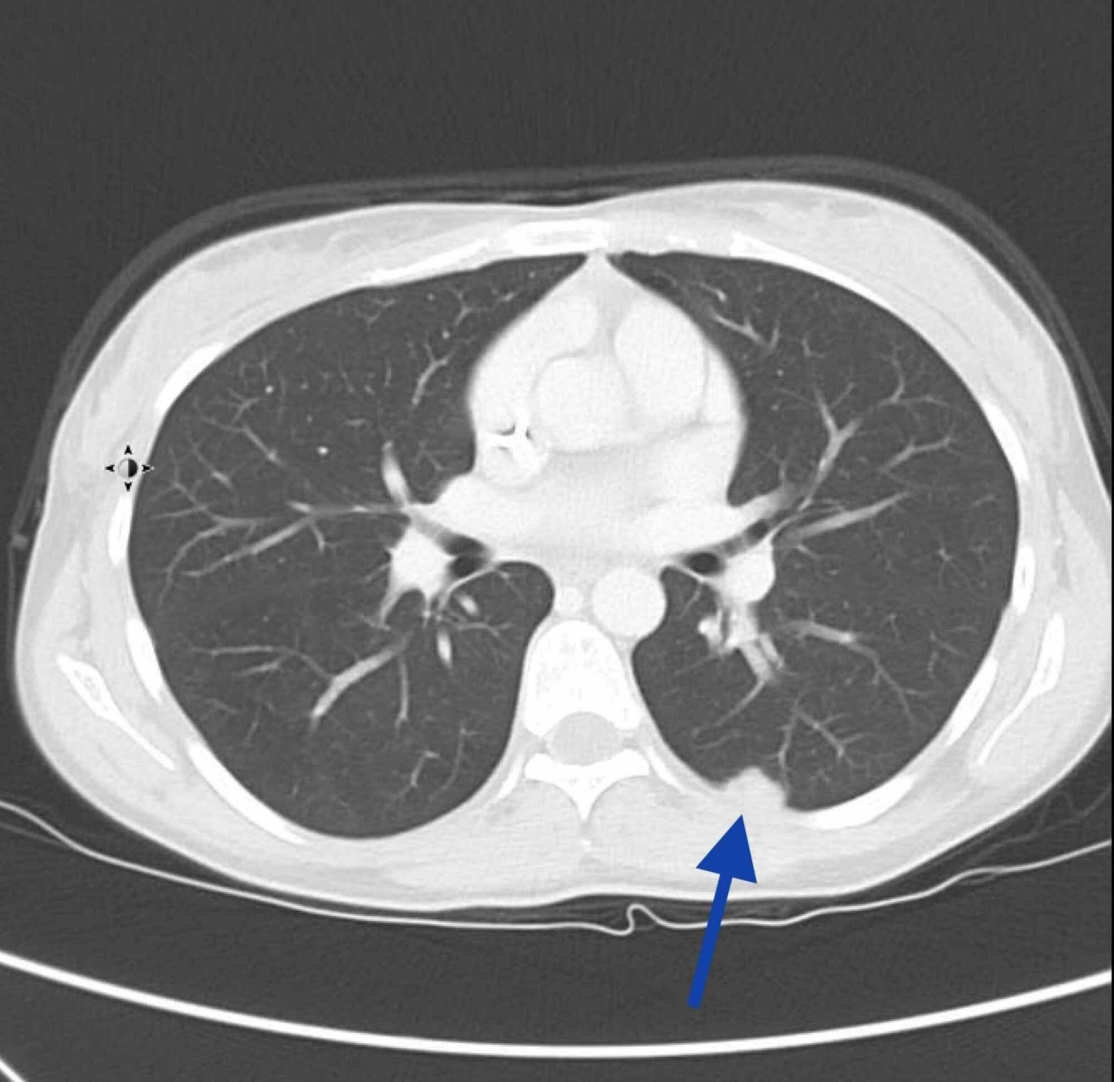
CT scan of axial section showing left lower lobe nodule (blue arrow)

**Figure 3:**
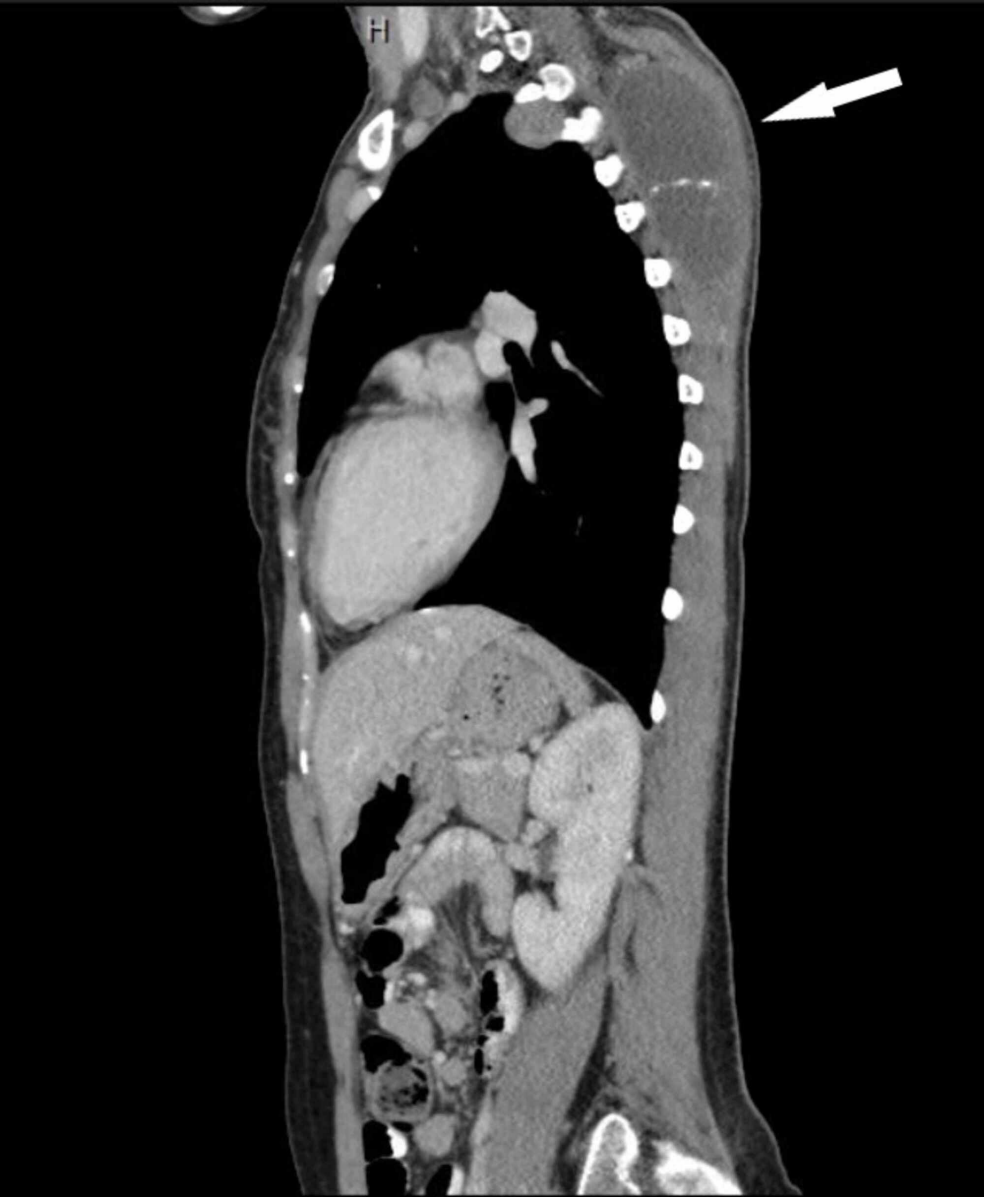
CT scan of sagittal section showing left trapezius muscle mass (white arrow)

She established care in our institution in February 2019. A repeat CT revealed a left pleural nodule measuring 3.9 cm, left lower lobe nodule measuring 1.5 cm, and a right upper lobe nodule measuring 3.5 cm (Figures 1, 2). The CT scan also revealed a trapezius mass measuring 8 x 4 cm (Figures 1, 3). She was not a candidate for resection of chest wall tumor due to the extensive nature of the required surgery. Pazopanib 800 mg daily was started due to disease progression, in addition to palliative radiation to the left scapula for pain control. Radiation was completed in May 2019. Clinically, her symptoms improved, and she had stable disease on imaging. She developed leukopenia during radiation therapy, which was attributed to medication side effects, so pazopanib was held in May and was resumed after completion of radiation therapy. A fluctuating thyroid-stimulating hormone level was observed and was attributed to drug-induced thyroiditis, with transient subclinical hyperthyroidism followed by hypothyroidism. This was treated with levothyroxine 75mcg.

**Figure 4:**
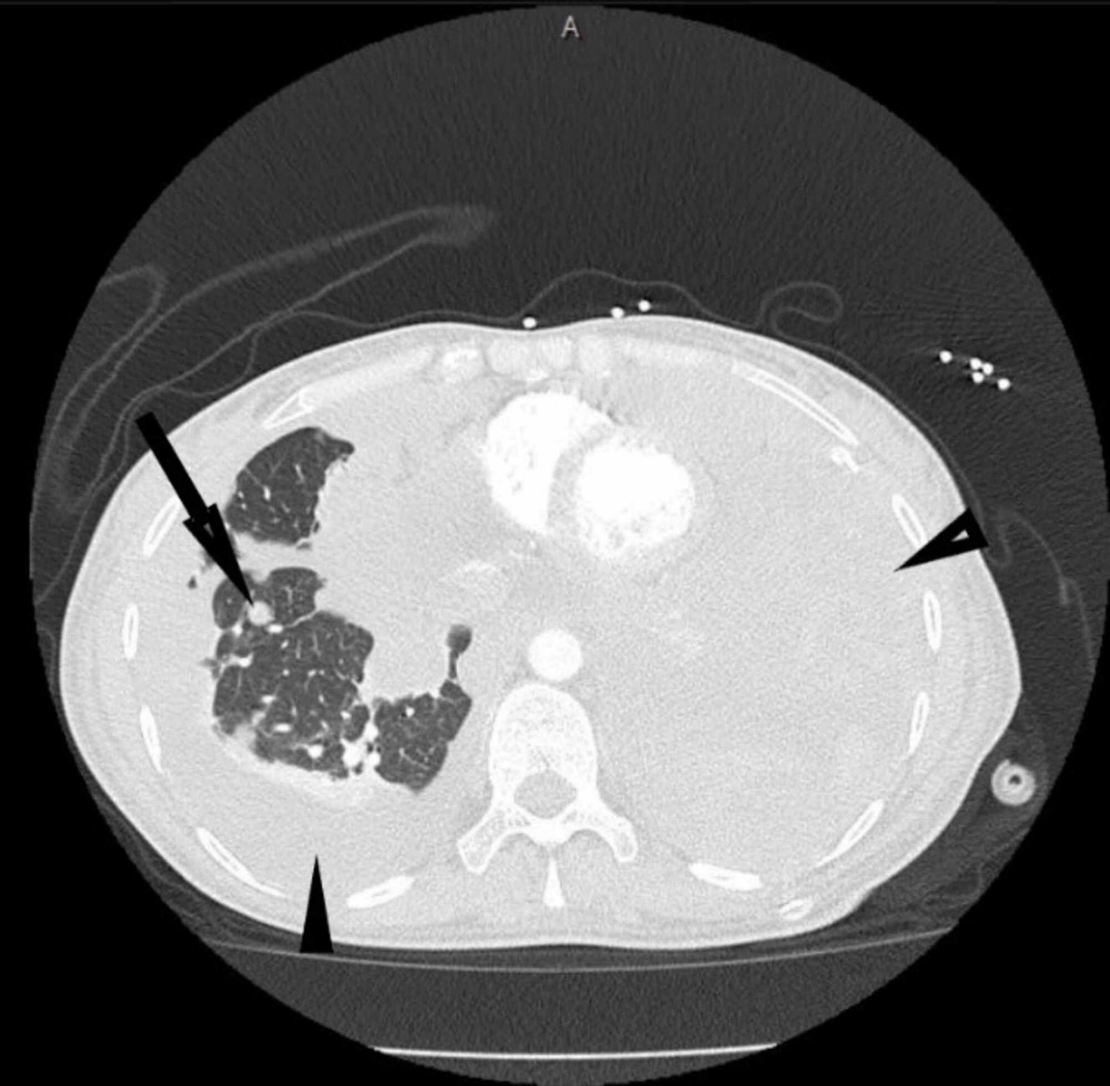
CT scan of axial section showing loculated malignant fluid collections compressing most of left lung and right lung base (arrowheads) with scattered lobular sarcomatous masses (arrow)

After a few months of treatment with pazopanib, she presented with acute shortness of breath and was diagnosed with spontaneous right-sided tension pneumothorax in October 2019, secondary to necrosis of metastatic lung lesions. This was possibly caused by pazopanib therapy, and therefore, pazopanib was stopped and pneumothorax was treated with chest tube placement followed by chemical pleurodesis for management of the hydrothorax that developed later. A few weeks later, she was readmitted to the hospital due to uncontrolled left upper extremity pain and acute shortness of breath secondary to a large recurrent left-sided pleural effusion, which was treated with a pleurx catheter. Dyspnea continued to worsen despite treatment. CT-thorax obtained at this time revealed widespread bilateral hemi-thoracic loculated malignant pleural effusion compressing most of the left lung and right lung base (Figure 4). The remaining functional aerated lung included the right apex and a segment of left lung apex. Scattered lobular sarcomatous masses were noted on the CT scan, throughout the lower right lung base (Figure 4). The malignant pericardial effusion was comparable to prior exams. Due to the poor prognosis and substantial clinical deterioration, the patient was transferred out of the intensive care unit (ICU) to pursue comfort measures. Unfortunately, our patient passed away a few days later due to respiratory failure secondary to malignant disease progression.

## Discussion

Synovial sarcoma is known to present predominantly in lower extremities [1,3,4], with the exception of a few atypical presentations such as ours. We present a case of synovial sarcoma of trapezius origin. The currently recommended first-line treatment for primary synovial sarcoma is surgical resection [2]. However, our patient was not considered a surgical candidate because her primary cancer had already metastasized to the lungs at the time of diagnosis making the tumor burden too large for a successful resection. Therefore, she was started on chemotherapy, with plans for subsequent radiation if she developed significant pain at the primary tumor site. Although the first chemotherapeutic regimen of adriamycin and ifosfamide with mesna led to an initial decrease in the size of the primary tumor, followed by stable disease, her disease did progress again shortly afterwards. This led to the decision to start her on pazopanib, along with palliative radiation of the left scapula to control pain.

Pazopanib is a small-molecule vascular endothelial growth factor inhibitor, with inhibitory activity against multiple targets including vascular endothelial growth factor receptors 1, 2, and 3, as well as platelet-derived growth factor receptors [8]. It was approved in 2012 to be used as a single agent in patients with metastatic advanced non-adipocytic soft-tissue sarcoma, who had failed previous chemotherapy regimens [8]. This drug was approved through a phase 3 trial on the basis of significantly increased progression-free survival in patients with advanced disease compared to those who received a placebo [8]. Our patient responded well to pazopanib as evidenced by a decrease in the size and hardness of the primary tumor in the left scapular region after treatment. However, a few months after starting pazopanib, she developed spontaneous right-sided tension pneumothorax likely secondary to underlying necrotic lung metastases. Pazopanib was thought to have caused the necrosis, and therefore, the medication was stopped.

Based on a compilation of case series, the incidence of secondary spontaneous pneumothorax (SSP) in sarcoma is only 1.9% and bilateral involvement is common [9]. Among the 20 different sarcoma cell types reported in the literature to be associated with SSP, synovial sarcoma appears to be the third most common, with an incidence of 8.5% [9]. The most common chest radiographic findings seen in patients who had sarcoma and later developed SSP include multiple nodules (48.4%) and cavitary or cystic lesions (25.8%) [9]. Although not proven, the suggested reason behind the incidence of SSP in people who have lung metastases and are on some form of chemo or targeted therapy is that cytotoxic agents may be inducing necrosis of lung nodules, thereby increasing the risk of rupture and development of pneumothorax [9,10]. The most common chemotherapeutic agents used before the occurrence of SSP were doxorubicin (49.1%), cyclophosphamide (37.6%) and vincristine (35.8%) [9]. However, two-thirds of the patients in this series developed pneumothorax before initiation of treatment [9], which questions the validity of the suggestion that these drugs directly contributed to the development of SSP. In those who got SSP after the initiation of chemotherapy, the median time duration between the two events was 90 days, with considerable variability across patients [9].

In our case, the drug that was thought to be responsible for triggering the SSP was pazopanib. There are individual case reports in the literature that report the incidence of SSP in sarcoma patients who had lung metastases, after beginning treatment with pazopanib [11,12]. However, the PALETTE phase 3 study, which included a total of 369 patients, and which eventually led to the approval of pazopanib in sarcoma, only detected a 3% pneumothorax incidence in the treatment group compared to 1% in the placebo group [8]. Also, there is no causal association established between pazopanib and incidence of SSP in the medical literature [10]. It is fairly evident from larger case-control studies and reviews that lung metastases, including pleural based nodules and cavitary lesions are independent risk factors for the incidence of SSP in sarcoma patients [9,10]. At least one pleural nodule was detected in our patient as early as July 2018 when her primary malignancy was first diagnosed, which then multiplied into widespread lung metastases over the course of her disease. These lesions could have independently culminated in her tension pneumothorax in October 2019. However, given the anti-angiogenic property of pazopanib [10], it is possible that this drug could have accelerated the necrosis of lung lesions that were already present at the time the drug was started at the beginning of the year 2019.

Overall, the prognosis of sarcoma-associated SSP is poor, and the one-year survival rate is generally around 20% [9]. To our knowledge, all published case reports of sarcoma patients who developed SSP after the initiation of pazopanib report that the drug was immediately terminated, and pneumothorax treated [11,12]. In cases where pazopanib was never re-initiated after the resolution of pneumothorax, the patients passed away due to disease progression within the next one to three months [11,12], similar to our patient.

## Conclusions

Through this case, we highlight the dilemma of attributing an unexpected complication like pneumothorax to therapeutic side effects versus an independent result of malignant disease progression. Pneumothorax in the setting of cancer with lung metastases worsens respiratory status and further decreases the chance of survival. Therefore, the clinical decision to continue a specific therapeutic agent in spite of complications that ensue, must be made by carefully weighing the beneficial effect of the drug in reducing further disease progression against validated side effects and the natural course a disease would take in the absence of treatment.
